# *N*-(1,3-Thia­zol-2-yl)-2-(2,4,6-trimethyl­phen­yl)acetamide

**DOI:** 10.1107/S1600536812031595

**Published:** 2012-07-18

**Authors:** Hoong-Kun Fun, Ching Kheng Quah, Prakash S. Nayak, B. Narayana, B. K. Sarojini

**Affiliations:** a X-ray Crystallography Unit, School of Physics, Universiti Sains Malaysia, 11800 USM, Penang, Malaysia; b Department of Studies in Chemistry, Mangalore University, Mangalagangotri 574 199, India; c Department of Chemistry, P. A. College of Engineering, Nadupadavu, Mangalore 574 153, India

## Abstract

In the title compound, C_14_H_16_N_2_OS, the thia­zole ring is essentially planar (r.m.s. deviation = 0.005 Å) and it forms a dihedral angle of 75.21 (8)° with the benzene ring. In the crystal, mol­ecules are linked into inversion dimers by pairs of N—H⋯N hydrogen bonds to generate *R*_2_^2^(8) loops.

## Related literature

For general background to and related structures of the title compound, see: Fun *et al.* (2011*a* ▶,*b* ▶, 2012 ▶). For graph-set notation of hydrogen bonds, see: Bernstein *et al.* (1995 ▶). For the stability of the temperature controller used for the data collection, see: Cosier & Glazer (1986 ▶).
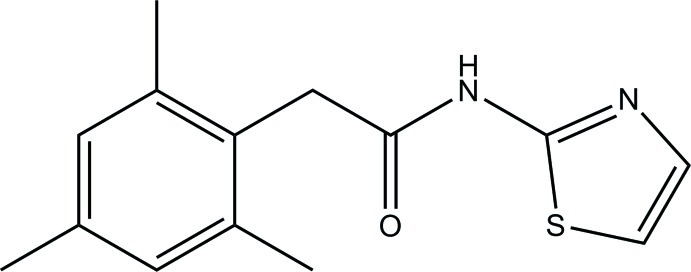


## Experimental

### 

#### Crystal data

C_14_H_16_N_2_OS
*M* *_r_* = 260.35
Monoclinic, 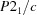

*a* = 16.421 (2) Å
*b* = 4.6397 (7) Å
*c* = 20.1144 (18) Å
β = 123.150 (7)°
*V* = 1283.1 (3) Å^3^
*Z* = 4
Mo *K*α radiation
μ = 0.24 mm^−1^
*T* = 100 K
0.30 × 0.25 × 0.09 mm

#### Data collection

Bruker SMART APEXII DUO CCD diffractometer
Absorption correction: multi-scan (*SADABS*; Bruker, 2009 ▶) *T*_min_ = 0.932, *T*_max_ = 0.979
13735 measured reflections
3748 independent reflections
2999 reflections with *I* > 2σ(*I*)
*R*_int_ = 0.040

#### Refinement

*R*[*F* ^2^ > 2σ(*F* ^2^)] = 0.040
*wR*(*F* ^2^) = 0.103
*S* = 1.03
3748 reflections
166 parameters
H-atom parameters constrained
Δρ_max_ = 0.39 e Å^−3^
Δρ_min_ = −0.51 e Å^−3^

### 

Data collection: *APEX2* (Bruker, 2009 ▶); cell refinement: *SAINT* (Bruker, 2009 ▶); data reduction: *SAINT*; program(s) used to solve structure: *SHELXTL* (Sheldrick, 2008 ▶); program(s) used to refine structure: *SHELXTL*; molecular graphics: *SHELXTL*; software used to prepare material for publication: *SHELXTL* and *PLATON* (Spek, 2009 ▶).

## Supplementary Material

Crystal structure: contains datablock(s) global, I. DOI: 10.1107/S1600536812031595/hb6891sup1.cif

Structure factors: contains datablock(s) I. DOI: 10.1107/S1600536812031595/hb6891Isup2.hkl

Supplementary material file. DOI: 10.1107/S1600536812031595/hb6891Isup3.cml

Additional supplementary materials:  crystallographic information; 3D view; checkCIF report

## Figures and Tables

**Table 1:** Hydrogen-bond geometry (Å, °)

*D*—H⋯*A*	*D*—H	H⋯*A*	*D*⋯*A*	*D*—H⋯*A*
N2—H1⋯N1^i^	0.88	2.08	2.9623 (19)	175

Symmetry code: (i) 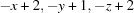.

**Fig. 1.**
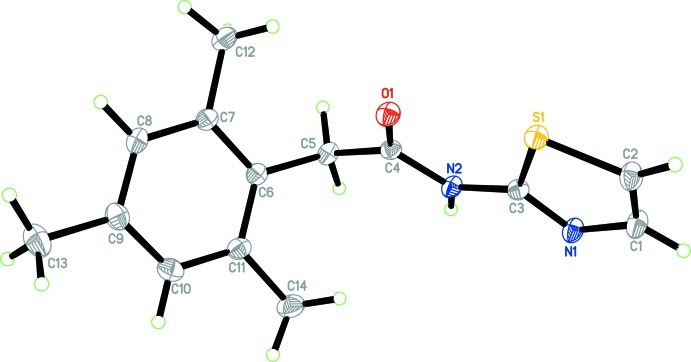
The molecular structure of the title compound showing 50% probability displacement ellipsoids for non-H atoms.

**Fig. 2.**
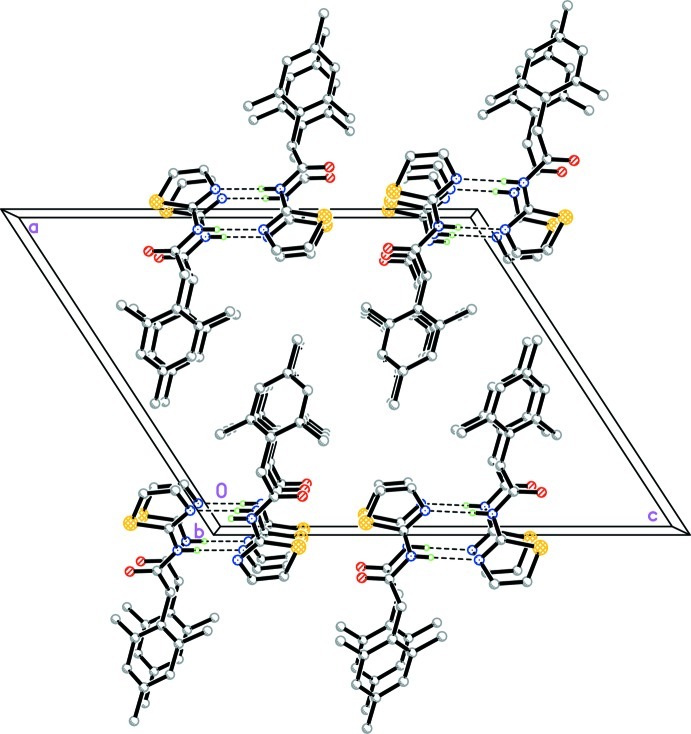
The crystal structure of the title compound, viewed along the b axis. H atoms not involved in hydrogen bonds (dashed lines) have been omitted for clarity.

## supplementary crystallographic information

### Comment

In continuation of our work on synthesis of amides (Fun *et al.*, 2011*a*,*b*, 2012), we report herein the crystal structure of the title compound.

In the title molecule (Fig. 1), the thiazol-2-yl ring (S1/N1/C1–C3) is essentially planar (r.m.s. deviation = 0.005 Å) and it forms a dihedral angle of 75.21 (8)° with the benzene ring (C6–C11). Bond lengths and angles are comparable to those in related structures (Fun *et al.*, 2011*a*,*b*, 2012).

In the crystal (Fig. 2), molecules are linked into an inversion dimer by pairs of intermolecular N2—H1···N1 hydrogen bonds (Table 1).

### Experimental

2,4,6-Trimethylphenylacetic acid (0.178 g, 1 mmol), 2-aminothiazole (0.1 g, 1 mmol) and 1-ethyl-3-(3-dimethylaminopropyl)-carbodiimide hydrochloride (1.0 g, 0.01 mol) were dissolved in dichloromethane (20 ml). The mixture was stirred in presence of triethylamine at 273 K for about 3 h. The contents were poured into 100 ml of ice-cold aqueous hydrochloric acid with stirring, which was extracted thrice with dichloromethane. Organic layer was washed with saturated NaHCO_3_ solution and brine solution, dried and concentrated under reduced pressure to give the title compound. Colourless blocks were grown from an acetone and toluene (1:1) solvent mixture by the slow evaporation method (m.p. 457–459 K).

### Refinement

N-bound hydrogen atom was located in a difference Fourier map and refined using a riding model with *U*_iso_(H) = 1.2 *U*_eq_(N) [N—H = 0.8794 Å]. The remaining H atoms were positioned geometrically and refined using a riding model with C—H = 0.95-0.99 Å and *U*_iso_(H) = 1.2 or 1.5*U*_eq_(C). A rotating-group model was applied for the methyl groups.

### Crystal data

**Table:**

C_14_H_16_N_2_OS	*F*(000) = 552
*M**_r_* = 260.35	*D*_x_ = 1.348 Mg m^−^^3^
Monoclinic, *P*2_1_/*c*	Mo *K*α radiation, λ = 0.71073 Å
Hall symbol: -P 2ybc	Cell parameters from 4087 reflections
*a* = 16.421 (2) Å	θ = 3.0–29.9°
*b* = 4.6397 (7) Å	µ = 0.24 mm^−^^1^
*c* = 20.1144 (18) Å	*T* = 100 K
β = 123.150 (7)°	Block, colourless
*V* = 1283.1 (3) Å^3^	0.30 × 0.25 × 0.09 mm
*Z* = 4	

### Data collection

**Table:**

Bruker SMART APEXII DUO CCD diffractometer	3748 independent reflections
Radiation source: fine-focus sealed tube	2999 reflections with *I* > 2σ(*I*)
Graphite monochromator	*R*_int_ = 0.040
φ and ω scans	θ_max_ = 30.1°, θ_min_ = 1.5°
Absorption correction: multi-scan (*SADABS*; Bruker, 2009)	*h* = −23→20
*T*_min_ = 0.932, *T*_max_ = 0.979	*k* = −6→6
13735 measured reflections	*l* = −26→28

### Refinement

**Table:**

Refinement on *F*^2^	Primary atom site location: structure-invariant direct methods
Least-squares matrix: full	Secondary atom site location: difference Fourier map
*R*[*F*^2^ > 2σ(*F*^2^)] = 0.040	Hydrogen site location: inferred from neighbouring sites
*wR*(*F*^2^) = 0.103	H-atom parameters constrained
*S* = 1.03	*w* = 1/[σ^2^(*F*_o_^2^) + (0.0444*P*)^2^ + 0.7659*P*] where *P* = (*F*_o_^2^ + 2*F*_c_^2^)/3
3748 reflections	(Δ/σ)_max_ = 0.001
166 parameters	Δρ_max_ = 0.39 e Å^−^^3^
0 restraints	Δρ_min_ = −0.51 e Å^−^^3^

### Special details

**Table:**

Experimental. The crystal was placed in the cold stream of an Oxford Cryosystems Cobra open-flow nitrogen cryostat (Cosier & Glazer, 1986) operating at 100.0 (1) K.
Geometry. All esds (except the esd in the dihedral angle between two l.s. planes) are estimated using the full covariance matrix. The cell esds are taken into account individually in the estimation of esds in distances, angles and torsion angles; correlations between esds in cell parameters are only used when they are defined by crystal symmetry. An approximate (isotropic) treatment of cell esds is used for estimating esds involving l.s. planes.
Refinement. Refinement of F^2^ against ALL reflections. The weighted R-factor wR and goodness of fit S are based on F^2^, conventional R-factors R are based on F, with F set to zero for negative F^2^. The threshold expression of F^2^ > 2sigma(F^2^) is used only for calculating R-factors(gt) etc. and is not relevant to the choice of reflections for refinement. R-factors based on F^2^ are statistically about twice as large as those based on F, and R-factors based on ALL data will be even larger.

### Fractional atomic coordinates and isotropic or equivalent isotropic displacement parameters (Å^2^)

**Table:**

	*x*	*y*	*z*	*U*_iso_*/*U*_eq_	
S1	1.03056 (3)	0.78584 (8)	0.83439 (2)	0.01686 (10)	
O1	0.86995 (7)	0.4661 (2)	0.75301 (6)	0.0180 (2)	
N1	1.06903 (9)	0.7448 (3)	0.97704 (7)	0.0176 (2)	
N2	0.93660 (8)	0.4568 (3)	0.88579 (7)	0.0151 (2)	
H1	0.9347	0.3871	0.9256	0.018*	
C1	1.13491 (11)	0.9354 (3)	0.97864 (9)	0.0206 (3)	
H1A	1.1835	1.0281	1.0262	0.025*	
C2	1.12628 (11)	0.9823 (3)	0.90878 (9)	0.0203 (3)	
H2A	1.1671	1.1057	0.9017	0.024*	
C3	1.01013 (10)	0.6518 (3)	0.90413 (8)	0.0145 (3)	
C4	0.86909 (10)	0.3723 (3)	0.80944 (8)	0.0146 (3)	
C5	0.79327 (10)	0.1587 (3)	0.79947 (8)	0.0166 (3)	
H5A	0.7985	0.1370	0.8506	0.020*	
H5B	0.8060	−0.0318	0.7847	0.020*	
C6	0.69101 (10)	0.2559 (3)	0.73607 (8)	0.0153 (3)	
C7	0.64731 (10)	0.1640 (3)	0.65713 (8)	0.0158 (3)	
C8	0.55387 (10)	0.2626 (3)	0.60037 (9)	0.0184 (3)	
H8A	0.5238	0.1988	0.5471	0.022*	
C9	0.50331 (11)	0.4500 (3)	0.61885 (9)	0.0201 (3)	
C10	0.54813 (11)	0.5407 (3)	0.69713 (9)	0.0211 (3)	
H10A	0.5147	0.6698	0.7109	0.025*	
C11	0.64099 (11)	0.4466 (3)	0.75590 (9)	0.0180 (3)	
C12	0.69872 (11)	−0.0344 (3)	0.63240 (9)	0.0209 (3)	
H12A	0.6596	−0.0557	0.5745	0.031*	
H12B	0.7076	−0.2234	0.6572	0.031*	
H12C	0.7623	0.0469	0.6494	0.031*	
C13	0.40305 (12)	0.5556 (4)	0.55574 (10)	0.0287 (4)	
H13A	0.3996	0.7646	0.5607	0.043*	
H13B	0.3546	0.4608	0.5624	0.043*	
H13C	0.3899	0.5098	0.5032	0.043*	
C14	0.68632 (12)	0.5548 (4)	0.83981 (9)	0.0251 (3)	
H14A	0.6451	0.7057	0.8406	0.038*	
H14B	0.7509	0.6340	0.8592	0.038*	
H14C	0.6923	0.3951	0.8741	0.038*	

### Atomic displacement parameters (Å^2^)

**Table:**

	*U*^11^	*U*^22^	*U*^33^	*U*^12^	*U*^13^	*U*^23^
S1	0.01899 (17)	0.02010 (18)	0.01301 (16)	−0.00305 (13)	0.00974 (14)	0.00054 (13)
O1	0.0200 (5)	0.0210 (5)	0.0134 (5)	−0.0015 (4)	0.0094 (4)	0.0009 (4)
N1	0.0188 (6)	0.0209 (6)	0.0125 (5)	−0.0052 (5)	0.0083 (5)	−0.0013 (4)
N2	0.0174 (6)	0.0170 (6)	0.0114 (5)	−0.0037 (4)	0.0082 (5)	−0.0004 (4)
C1	0.0201 (7)	0.0230 (8)	0.0167 (7)	−0.0076 (6)	0.0087 (6)	−0.0021 (6)
C2	0.0192 (7)	0.0230 (7)	0.0183 (7)	−0.0054 (6)	0.0099 (6)	0.0001 (6)
C3	0.0164 (6)	0.0145 (6)	0.0136 (6)	0.0002 (5)	0.0089 (5)	0.0007 (5)
C4	0.0160 (6)	0.0134 (6)	0.0139 (6)	0.0019 (5)	0.0077 (5)	0.0002 (5)
C5	0.0185 (7)	0.0151 (7)	0.0144 (6)	−0.0022 (5)	0.0079 (5)	0.0004 (5)
C6	0.0169 (6)	0.0133 (6)	0.0161 (6)	−0.0020 (5)	0.0092 (5)	0.0002 (5)
C7	0.0182 (6)	0.0125 (6)	0.0168 (6)	−0.0024 (5)	0.0097 (6)	−0.0009 (5)
C8	0.0181 (7)	0.0188 (7)	0.0155 (7)	−0.0037 (5)	0.0074 (6)	−0.0013 (5)
C9	0.0176 (7)	0.0211 (7)	0.0221 (7)	0.0000 (5)	0.0111 (6)	0.0034 (6)
C10	0.0217 (7)	0.0220 (8)	0.0240 (8)	0.0012 (6)	0.0155 (6)	0.0001 (6)
C11	0.0210 (7)	0.0176 (7)	0.0186 (7)	−0.0032 (5)	0.0130 (6)	−0.0020 (5)
C12	0.0245 (7)	0.0178 (7)	0.0169 (7)	0.0015 (6)	0.0091 (6)	−0.0020 (5)
C13	0.0211 (8)	0.0375 (10)	0.0267 (8)	0.0061 (7)	0.0125 (7)	0.0083 (7)
C14	0.0294 (8)	0.0288 (9)	0.0211 (8)	−0.0019 (7)	0.0164 (7)	−0.0051 (6)

### Geometric parameters (Å, º)

**Table:**

S1—C2	1.7227 (16)	C7—C12	1.506 (2)
S1—C3	1.7267 (14)	C8—C9	1.386 (2)
O1—C4	1.2230 (17)	C8—H8A	0.9500
N1—C3	1.3119 (18)	C9—C10	1.389 (2)
N1—C1	1.3840 (19)	C9—C13	1.509 (2)
N2—C4	1.3709 (18)	C10—C11	1.394 (2)
N2—C3	1.3875 (18)	C10—H10A	0.9500
N2—H1	0.8794	C11—C14	1.510 (2)
C1—C2	1.351 (2)	C12—H12A	0.9800
C1—H1A	0.9500	C12—H12B	0.9800
C2—H2A	0.9500	C12—H12C	0.9800
C4—C5	1.516 (2)	C13—H13A	0.9800
C5—C6	1.519 (2)	C13—H13B	0.9800
C5—H5A	0.9900	C13—H13C	0.9800
C5—H5B	0.9900	C14—H14A	0.9800
C6—C7	1.404 (2)	C14—H14B	0.9800
C6—C11	1.405 (2)	C14—H14C	0.9800
C7—C8	1.397 (2)		
			
C2—S1—C3	88.58 (7)	C9—C8—H8A	118.7
C3—N1—C1	108.98 (12)	C7—C8—H8A	118.7
C4—N2—C3	122.51 (12)	C8—C9—C10	118.04 (14)
C4—N2—H1	120.3	C8—C9—C13	121.07 (14)
C3—N2—H1	117.2	C10—C9—C13	120.89 (15)
C2—C1—N1	116.28 (14)	C9—C10—C11	121.49 (14)
C2—C1—H1A	121.9	C9—C10—H10A	119.3
N1—C1—H1A	121.9	C11—C10—H10A	119.3
C1—C2—S1	110.20 (11)	C10—C11—C6	119.66 (13)
C1—C2—H2A	124.9	C10—C11—C14	119.06 (14)
S1—C2—H2A	124.9	C6—C11—C14	121.28 (14)
N1—C3—N2	120.93 (12)	C7—C12—H12A	109.5
N1—C3—S1	115.96 (11)	C7—C12—H12B	109.5
N2—C3—S1	123.11 (10)	H12A—C12—H12B	109.5
O1—C4—N2	121.68 (13)	C7—C12—H12C	109.5
O1—C4—C5	122.36 (13)	H12A—C12—H12C	109.5
N2—C4—C5	115.95 (12)	H12B—C12—H12C	109.5
C4—C5—C6	111.57 (12)	C9—C13—H13A	109.5
C4—C5—H5A	109.3	C9—C13—H13B	109.5
C6—C5—H5A	109.3	H13A—C13—H13B	109.5
C4—C5—H5B	109.3	C9—C13—H13C	109.5
C6—C5—H5B	109.3	H13A—C13—H13C	109.5
H5A—C5—H5B	108.0	H13B—C13—H13C	109.5
C7—C6—C11	119.71 (13)	C11—C14—H14A	109.5
C7—C6—C5	120.39 (13)	C11—C14—H14B	109.5
C11—C6—C5	119.87 (13)	H14A—C14—H14B	109.5
C8—C7—C6	118.59 (13)	C11—C14—H14C	109.5
C8—C7—C12	119.60 (13)	H14A—C14—H14C	109.5
C6—C7—C12	121.81 (13)	H14B—C14—H14C	109.5
C9—C8—C7	122.52 (14)		
			
C3—N1—C1—C2	−0.6 (2)	C5—C6—C7—C8	−178.83 (13)
N1—C1—C2—S1	0.76 (19)	C11—C6—C7—C12	178.50 (13)
C3—S1—C2—C1	−0.54 (13)	C5—C6—C7—C12	0.6 (2)
C1—N1—C3—N2	179.97 (13)	C6—C7—C8—C9	0.8 (2)
C1—N1—C3—S1	0.13 (17)	C12—C7—C8—C9	−178.67 (14)
C4—N2—C3—N1	174.78 (13)	C7—C8—C9—C10	−0.2 (2)
C4—N2—C3—S1	−5.40 (19)	C7—C8—C9—C13	179.08 (15)
C2—S1—C3—N1	0.23 (13)	C8—C9—C10—C11	−0.3 (2)
C2—S1—C3—N2	−179.60 (13)	C13—C9—C10—C11	−179.52 (15)
C3—N2—C4—O1	−0.3 (2)	C9—C10—C11—C6	0.1 (2)
C3—N2—C4—C5	−179.76 (13)	C9—C10—C11—C14	179.44 (14)
O1—C4—C5—C6	−49.20 (19)	C7—C6—C11—C10	0.5 (2)
N2—C4—C5—C6	130.23 (13)	C5—C6—C11—C10	178.43 (13)
C4—C5—C6—C7	92.65 (16)	C7—C6—C11—C14	−178.81 (14)
C4—C5—C6—C11	−85.24 (16)	C5—C6—C11—C14	−0.9 (2)
C11—C6—C7—C8	−0.9 (2)		

### Hydrogen-bond geometry (Å, º)

**Table:**

*D*—H···*A*	*D*—H	H···*A*	*D*···*A*	*D*—H···*A*
N2—H1···N1^i^	0.88	2.08	2.9623 (19)	175

Symmetry code: (i) −*x*+2, −*y*+1, −*z*+2.
